# An Analysis on the Factors for Cervical Insufficiency Causing Adverse Emotions Among Pregnant Women at Different Gestation Phases

**DOI:** 10.3389/fpsyt.2022.764345

**Published:** 2022-04-06

**Authors:** Qichang Wu, Jiebing Chen, Qiaojian Zou, Xun Zeng, Yan Yang, Yijia Zhou, Guimei He, Chunqi Luo, Fengchun Wu

**Affiliations:** ^1^Department of Outpatient, The First Affiliated Hospital of Sun Yat-sen University, Guangzhou, China; ^2^Department of Obstetrics and Gynecology, The First Affiliated Hospital of Sun Yat-sen University, Guangzhou, China; ^3^Department of Psychiatry, The Affiliated Brain Hospital of Guangzhou Medical University, Guangzhou, China; ^4^Guangdong Engineering Technology Research Center for Translational Medicine of Mental Disorders, Guangzhou, China

**Keywords:** gestation phase, cervical insufficiency, adverse emotions, anxiety, depression

## Abstract

**Background:**

To analyze the anxiety, depression, and related factors among pregnant women with cervical insufficiency, so as to provide a reference for clinical psychological intervention as an adjuvant therapy.

**Methods:**

A total of 101 cases in China with cervical insufficiency were included in the observation group by a convenience sampling method, and 114 normal healthy women of childbearing age were selected as the control group. Participants were investigated and observed for anxiety and depression by SAS and SDS, respectively, to analyze the emotional state and influencing factors of the patients with cervical insufficiency. Stratified by the first, second and third trimesters, our study used whether depressive or not and whether anxiety or not as the dichotomous variables. A multivariate Logistic regression was adopted to analyze the influencing factors. Relevant influencing factors were screened out by the forward stepwise method in combination with professional knowledge and the number of variables.

**Results:**

There were statistical significant differences in SAS and SDS between observation group and control group and the incidence rate of anxiety and depression was higher in pregnant women with CI. Multivariate Logistic regression demonstrated that history of abnormal pregnancy was the main influencing factor for anxiety and depression in the early and middle gestation phases, and cervical insufficiency was the factor influencing the anxiety in early gestation and both anxiety and depression in the late gestation phase.

**Conclusion:**

Cervical insufficiency may have a negative impact on the emotions of pregnant women. Individualized and targeted mental care should be added into clinic work to prevent negative outcomes.

## Introduction

Cervical insufficiency (CI) refers to an anatomical or functional abnormality causing progressive and painless shortened, dilated, flattened, and funneled cervix before full-term pregnancy, which may lead to the inability to maintain pregnancy in the 2nd and 3rd trimesters. CI accounts for about 1% of all pregnancies and it is an important reason for recurrent abortion and premature delivery in the 2nd and 3rd trimesters (1). Previous studies have shown that unwell in gabortions caused by CI mainly occurs between 18 and 25 weeks of gestation and it accounts for about 15% of recurrent abortions between 16 and 28 weeks (2). The pregnancy loss at this stage may cause irreversible damage to the body, and even to the mind (3, 4).

By now, several surgical methods have been proposed for the treatment of CI worldwide, including transvaginal and transabdominal cervical cerclages, in which the latter can be done by open or laparoscopic surgery (1, 5). Yao et al. have found that laparoscopic cervical cerclages can increase the gestational weeks for patients with CI, thus bettering the pregnancy outcomes (6).

During the pregnancy period, women would obtain greatly change not only in their physiological, but also their psychological state. Previously studies demonstrated that up to 11.9% of pregnancy women could be diagnosis as depression state (7), meanwhile 22.9% of pregnant women suffer from anxiety (8, 9). In China, it is reported that about 19.7% of women suffer from antenatal depression (10), while 34%of pregnant women have anxiety syndrome (11). Depression and anxiety disorders during pregnancy can lead to serious consequence to women, infants, and also their families. In some cases, those patients even come up with commit suicide or infanticide, leading to extremely bad impact on family and society (12).

As we mentioned above, patients with CI may have several times of unwilling spontaneous abortion between 16 and 28 weeks. Study have shown that unwilling pregnancy termination before 28 weeks of gestation, which often happen in patients suffered from CI, could lead to adverse mental health consequences (13). Since women can build up a deep relationship with their babies by fetal heartbeat and fetal movement in the 2nd trimester, pregnancy loss at this stage can cause graver damage. Besides, recurrent abnormal pregnancies and the increasing age can cause great psychological pressure on those patients among their daily lives and their next entire pregnancies (13, 14). As the 2nd and 3rd trimesters of pregnancy is the critical period for pregnant women with CI, understanding the physiological and emotional changes of pregnant women with CI after laparoscopic cervical cerclage may help to find problems early and solve them in time, thus obtaining good pregnancy outcomes. However, there is still no study on the adverse emotions of pregnant women with CI during pregnancy or the changes in psychological state of pregnant women after laparoscopic cervical cerclage.

In our study, we tried to explore the emotional state of patients with CI at different gestation phases and relevant influencing factors for the first time in China, so as to provide specific health education for pregnant women with CI, improve their psychological state, promote maternal and child safety, and provide valuable reference for clinical intervention and nursing.

## Methods

### Source of Data

A total of 101 pregnant women diagnosed as CI in the First Affiliated Hospital of Sun Yat-sen University (Guangzhou, China) between May 2019 and December 2020 were included as the observation group by a convenient sampling method, and 114 healthy women of childbearing age were included as the control group. Inclusion criteria for the observation group: ① Patients with definite CI; ② Patients who received laparoscopic cervical cerclage during early pregnancy in our hospital; ③ Patients with intrauterine pregnancy confirmed by B-ultrasonography, gestational weeks ≤15; ④ Those understanding the investigation and willing to cooperate; ⑤ With live fetus confirmed before surgery. Based on a principle of randomness and equilibrium, 114 healthy pregnant women in the same period were included as the control group. Inclusion criteria for the control group: ① Healthy women of childbearing age, aged 18–49 years; ② Pregnant women without chronic diseases and pregnancy-related complications;③ Those voluntarily cooperating with the investigators. Women who meet the following exclusion criteria will not be included: (1) People with mental retardation that may affect their understanding for the questionnaires; (2) People with history of mental illness and malignant tumor; (3) Those taking antipsychotic drugs during pregnancy; (4) Those with genetic diseases. In strict accordance with the inclusion and exclusion criteria and following the consent of the ethics committee, 116 CI patients signed on the informed consent, and 101 were finally included as observation group according to the criteria.

### Methods

#### Survey Methods

There was a psychological questionnaire during pregnancy, which was composed of general information and scales for psychological testing of anxiety and depression. The questionnaire survey is conducted by a face-to-face interview for the investigators collecting relevant data one by one. After the investigators answer the questionnaire in detail, the assessed subjects fill in the questionnaire independently. The subjects completed the questionnaires independently after the investigators explained in detail, and then, the investigators collected the questionnaires and check them one by one, so as to guarantee the authenticity and validity of the survey results.

All participants were fully informed and agreed to participate in this study. Written informed consent was obtained from all of the participants.

#### Assessment Method for Emotional Disorders

Self-rating Anxiety Scale (SAS) (15) and Self-rating Depression Scale (SDS) (16) were used to evaluate the patients' anxiety and depression, respectively, according to a great many of articles publish in the world and the expert consensus of Chinese doctors. Many published studies had demonstrated the good psychometric property and validity of both SAS (17, 18) and SDS (19–21). Both SAS and SDS scale consist of 20 items with 4 Likert levels, i.e., “none or seldom,” “a little time,” “considerable time,” “for the most part or all times.” The total score is the sum of all scores multiplied by 1.25, which is positively correlated with anxiety and depression. In exactly, total score lower than 50 is considered normal, while SAS score more than 50 can be diagnosed as anxiety, and can be divided into mildly anxiety (50–59), moderately anxiety (60–69), and severely anxiety (>69). For SDS, those have a score more than 53 can be diagnosed as depression, which has a degree of mildly depressed (53–62), moderately depressed (63–72) and severely depressed (>72).

### Statistical Analysis

Stratified by the first, second and third trimesters, our study used whether depressive or not and whether anxiety or not as the dichotomous variables. A multivariate Logistic regression was adopted to analyze the influencing factors. Relevant influencing factors were screened out by the forward stepwise method in combination with professional knowledge and the number of variables. The SPSS20.0 software was used for relevant statistical analysis. If *P* < 0.05, there was statistical significance for relevant difference. The internal consistency of current study data of SDS and SAS was calculated using SPSS20.0, and the result was α = 0.875.

### Statement of Ethics

This study has been approved by the Ethics Committee of the First Affiliated Hospital of Sun Yat-sen Medical University, approval number: Lunzhen (2020) No. 178.

## Results

The demographic data of the included patients are as shown in Table 1. We can notice that 77.23% patients in CI group had more than 2 times of pregnancy experience, as the same number in control group is 32.46%. Meanwhile, 90.10% of patients in CI group had the history of abnormal pregnancy of delivery, while only 40.35% of patients in control group have suffered the same, which is agree with the situation of cervical insufficiency.

**Table 1:** General information of the included patients.

**Variable**	**Control group (*n* = 114)**	**CI group (*n* = 101)**
Age (years)	32.08 ± 4.62	31.7 ± 4.13
Level of degree of education		
Primary school or below	3 (2.63)	3 (2.97)
Secondary school or technical	14 (12.28)	46 (45.54)
secondary school		
College degree or above	97 (85.09)	52 (51.49)
Gravidity		
1~	37 (32.46)	4 (3.96)
2-	40 (35.09)	19 (18.81)
3 or more	37 (32.46)	78 (77.23)
Parity		
0	53 (46.49)	53 (52.48)
1	58 (50.88)	36 (35.64)
2 or more	3 (2.63)	12 (11.88)
Child		
Without	58 (50.88)	63 (62.38)
With	56 (49.12)	38 (37.62)
History of abnormal pregnancy of delivery		
Without	68 (59.65)	10 (9.90)
With	46 (40.35)	91 (90.10)
History of induced abortion		
Without	81 (71.05)	62 (61.39)
With	33 (28.95)	39 (38.61)
History of spontaneous abortion		
Without	88 (77.19)	24 (23.76)
With	26 (22.81)	87 (86.14)

As shown in Table 2, the SAS and SDS scores among pregnant women with CI were significantly higher than healthy pregnant women no matter in the first, second or third trimester of pregnancy (*P* < 0.05). Meanwhile, no matter mild, moderately or severely anxiety and depression, we noticed the incidence rate was significantly increased in CI group from Table 3.

**Table 2:** Analysis on anxiety and depression during different trimesters.

**Group**	**SAS**			**SDS**		
	**Healthy pregnant women**	**Pregnant women with CI**	***P*-value**	**Healthy pregnant women**	**Pregnant women with CI**	***P*-value**
The 1st trimester	40.79 ± 6.91	43.44 ± 8.44	0.012	46.05 ± 8.23	51.30 ± 10.37	0.001
The 2nd trimester	38.19 ± 6.78	43.43 ± 10.23	0.001	43.64 ± 9.22	48.75 ± 12.33	0.001
The 3rd trimester	38.24 ± 5.99	42.00 ± 9.22	0.001	42.25 ± 8.52	47.07 ± 11.06	0.001

**Table 3:** Incident rate of anxiety and depression between two groups.

**Group**	Anxiety^*^		Depression^*^	
	**Healthy pregnant women**	**Pregnant women with CI**	**Healthy pregnant women**	**Pregnant women with CI**
The 1st trimester	11 (9.64%)	19 (18.81%)	18 (15.79%)	48 (47.52%)
The 2nd trimester	7 (6.14%)	22 (21.78%)	20 (17.54%)	43 (42.57%)
The 3rd trimester	3 (2.63%)	15 (14.85%)	11 (9.65%)	35 (34.65%)

* *Anxiety was diagnosis by SAS score higher than 50 and depression was diagnosis by SDS score higher than 53 as mentioned in methods part*.

As shown in Tables 4, 5, the results of multivariate logistic regression analysis are as follows: in the 1st trimester, the history of abnormal pregnancy is the factor for the occurrence of anxiety, while the history of abnormal pregnancy and CI are the factors for the occurrence of depression; in the 2nd trimester, the history of abnormal pregnancy is the factor for the occurrence of anxiety, while the history of abnormal pregnancy and history of induced abortion are the factors for the occurrence of depression; in the third trimester, CI is the factor for the occurrence of anxiety, while the gravidity and CI are the factors for the occurrence of depression.

**Table 4:** Multivariate regression analysis on the anxiety during pregnancy.

**Trimester**	**Factor**	**B**	**OR**	**95%CI**	***P***
The 1st trimester	History of abnormal pregnancy	2.278	9.761	2.258–42.207	0.002
The 2nd trimester	History of abnormal pregnancy	2.287	9.841	2.273–42.616	0.002
The 3rd trimester	CI	1.925	6.852	1.920–24.453	0.003

**Table 5:** Multivariate regression analysis on the depression during pregnancy.

**Trimester**	**Factor**	**B**	**OR**	**95%CI**	***P***
The 1st trimester	History of abnormal pregnancy	2.326	10.238	3.356–31.232	<0.001
	CI	0.800	2.224	1.095–4.518	0.027
The 2nd trimester	History of abnormal pregnancy	2.318	10.158	3.822–26.996	<0.001
	History of induced abortion	0.974	2.648	1.362–5.150	0.004
The 3rd trimester	Parity	0.381	1.464	1.096–1.954	0.010
	CI	1.118	3.060	1.302–7.189	0.010

## Discussion

Muyiduli et al. (22) and Bunevicius et al. (23) found that the incidence rates of anxiety and depression in the 1st trimester was significantly higher than in the 2nd or 3rd trimesters, which was generally consistent with the results of our study, but by now, there has been no study on the psychological state of pregnant women with CI. Our study found that although the pregnant women in the CI group had undergone laparoscopic cervical cerclage before pregnancy or in the 1st trimester, there were still 18.8 and 47.5% of them with different degrees of anxiety and depression in the 1st trimester, which were significantly higher than 9.6 and 15.79%, the incidence rates of anxiety and depression in the healthy pregnant women. At the same time, a comparison of SAS and SDS scores found that the depression of pregnant women with CI was improved with the increase of gestational weeks, but the anxiety continued throughout the whole pregnant period. Maybe the influencing factors for anxiety and depression varies with psychological states of the pregnant women with CI. As a natural law, the pregnancy period cannot be changed. This study explored the influencing factors based on three trimesters, namely the 1st, 2nd, and 3rd trimesters. The results showed that it was feasible to have a specific intervention after finding the influencing factors of anxiety and depression at different trimesters. The psychological stress of pregnant women with CI in the observation group increased with the growth of their babies. In the 2nd and 3rd trimesters, as the feelings between the mothers and their babies are stronger and stronger, the history of abnormal pregnancy may increase the mothers' psychological burden (4). They fear losing their children again, and are swayed by considerations of gain and loss. This is bad for their physical and mental health. Even the slightest hint may make the expectant mothers nervous (3, 4). After the peri-dangerous weeks (before and after the past weeks of spontaneous abortion), the depression of pregnant women in the control group was gradually improved until safe delivery.

This study found that among pregnant women with CI, history of abnormal pregnancy or delivery was the main factor influencing the anxiety and depression in the first and second trimesters, and CI was the factor influencing the anxiety in the first trimester as well as the anxiety and depression in the third trimester. CI may cause poor pregnancy outcomes, and history of repeated abortion in the 2nd trimester of pregnancy may cause endless harm to both the pregnant women and their families (13). According to relevant literature, the incidence rate of preterm delivery among patients with CI is about 3.3 times that among healthy pregnant women, and furthermore, 8–9% of preterm delivery cases, 40–50% of natural preterm delivery cases, and 20–30% of cases with premature rupture of membranes are caused by CI (24). Spontaneous abortion, as a major negative event in life, may increase the psychological burden of relevant women, reduce their interest in other activities in their life, and form a barrier to contact with society for them, thus leading to their interpersonal tension and indifference, lowering their self-esteem, and increasing their frustration, loneliness and guilt (12). In this study, most of the pregnant women came to our hospital for help after several spontaneous abortions, and some of them even had five abortions in the 2nd trimester. They had been in a negative psychological stimulation for a long time, and so, they were eager to get pregnant, but at the same time, they were afraid of losing their babies again. In addition, maternal anxiety and depression may also affect their children's behavior in the future. Previous studies have shown that mothers' perinatal depression is associated with depression, social withdrawal, impulsivity, and aggression of their children (25, 26). Therefore, early identification of CI and adverse emotions, as well as early diagnosis and timely psychological intervention, is extremely necessary. Formulating health education plan in clinical practice and the implementing individualized nursing care can effectively relieve the adverse emotions of pregnant women in the observation group, thus promoting the health of both mothers and children.

Based on the characteristics of psychological conditions of the pregnant women with CI in different trimesters, an initial targeted intervention was carried out. We established a We Chat group named as “Sun Yat-sen - Circle of Life”, and arranged professional doctors to answer relevant questions and conduct health education in the group, so as to relieve the anxiety and depression of relevant women during pregnancy. However, this study is merely based on the authors' experience in remote education (27), and relevant interventions were also limited to some extent for lack of relevant experience. Moreover, online communication depends on when the members of research group are online. Maybe pregnant women with CI cannot receive response or guidance timely in case of an emergency. In the future, we will arrange online communication twice a week, so as to reply relevant questions in the circle as soon as possible, and carry out online outpatient service of experts, so as to provide professional guidance and answer relevant questions face to face. Additionally, we may organize relevant information from the communication of “Sun Yat-sen- Circle of Life” to formulate pamphlets and teaching videos for high-frequency problems, and have a simulated field display for possible emergencies during pregnancy, thus improving relevant health education through images and texts.

Previously researches didn't investigate the depression and anxiety situation in pregnancy women with cervical insufficiency. For the first time in the world, our study focus on the depression and anxiety in those patients after cervical cerclage, which can provide more helps on the care of this group. In addition, this article provides the knowledge of psychological nursing in pregnancy women with cervical insufficiency, which helps to prevent adverse pregnancy outcomes.

This study also has some limitations as follows: 1. Asthe self-rating scale was adopted in this study, unavoidable bias may occur since the subjectivity of the respondents. In future, examiner-rating scales should be used for further evaluation. 2. In this study, only patients with CI in the First Affiliated Hospital of Sun Yat-sen University from China were included, and so, the conclusion is to be further verified by multi-center studies.

## Conclusion

In conclusion, for the first time in the world, this study explored the emotional problems of pregnant women with CI. CI may affect the emotions of pregnant women, and poor psychological states may threaten the maternal and infant health in China. Individualized and targeted mental care should be added into clinic work to prevent negative outcomes. Further research can improve the credibility of research results by expanding the sample size and adopting more objective scales for evaluation.

## Data Availability Statement

The original contributions presented in the study are included in the article/supplementary material, further inquiries can be directed to the corresponding authors.

## Ethics Statement

The studies involving human participants were reviewed and approved by the First Affiliated Hospital of Sun Yat-sen University. The patients/participants provided their written informed consent to participate in this study.

## Author Contributions

FW and JC were responsible for management and oversight of the study. QW and QZ were responsible for general omnibus data analyses and were keys contributing authors to the manuscript. CL and GH were responsible for all research interviews and clinical chart reviews associated with this study. XZ and YZ provided guidance on the design of primary analyses. YY assisted with all data collection, analysis, and writing of the manuscript. All authors contributed to the study design and data interpretation and critically reviewed the manuscript and gave final approval for its publication.

## Funding

This work was supported by grants from the National Natural Science Foundation of China (No. 31771074), the Science and Technology Plan Project of Guangdong (No. 2019B030316001), the Program of Guangzhou (No. 201807010064), and the Science and Technology Plan of Xizang (No. XZ2019ZRG-152).

## Conflict of Interest

The authors declare that the research was conducted in the absence of any commercial or financial relationships that could be construed as a potential conflict of interest.

## Publisher's Note

All claims expressed in this article are solely those of the authors and do not necessarily represent those of their affiliated organizations, or those of the publisher, the editors and the reviewers. Any product that may be evaluated in this article, or claim that may be made by its manufacturer, is not guaranteed or endorsed by the publisher.
